# Polyvinyl Butyral Polymeric Host Material-Based Fluorescent Thin Films to Achieve Highly Efficient Red and Green Colour Conversion for Advanced Next-Generation Displays

**DOI:** 10.3390/nano13061009

**Published:** 2023-03-10

**Authors:** Ashish Gaurav, Yi-Shan Lin, Chih-Yuan Tsai, Jung-Kuan Huang, Ching-Fuh Lin

**Affiliations:** 1 Graduate Institute of Photonics and Optoelectronics, National Taiwan University, No. 1, Sec. 4, Roosevelt Road, Taipei 106319, Taiwan; 2 Graduate Institute of Electronics Engineering, National Taiwan University, No. 1, Sec. 4, Roosevelt Road, Taipei 106319, Taiwan; 3 Department of Electrical Engineering, National Taiwan University, No. 1, Sec. 4, Roosevelt Road, Taipei 106319, Taiwan

**Keywords:** DCJTB, coumarin-6, PVB, chromophore, concentration quenching, fluorescent dye, phosphor

## Abstract

Rare-earth element-free fluorescent materials are eco-friendlier than other traditional fluorescent precursors, such as quantum dots and phosphors. In this study, we explore a simple and facile solution-based technique to prepare fluorescent films, which are highly stable under ordinary room conditions and show hydrophobic behaviour. The proposed hybrid material was designed with hybrid composites that use polyvinyl butyral (PVB) as a host doped with organic dyes. The red and green fluorescent films exhibited quantum yields of 89% and 80%, respectively, and both are very uniform in thickness and water resistant. Additionally, PVB was further compared with another polymeric host, such as polyvinylpyrrolidone (PVP), to evaluate their binding ability and encapsulation behaviour. Next, the effect of PVB on the optical and chemical properties of the fluorescent materials was studied using UV spectroscopy and Fourier transform infrared spectroscopy. The analysis revealed that no new bond was formed between the host material and fluorescent precursor during the process, with intermolecular forces being present between different molecules. Moreover, the thickness of the fluorescent film and quantum yield relation were evaluated. Finally, the hydrophobic nature, strong binding ability, and optical enhancement by PVB provide a powerful tool for fabricating a highly efficient fluorescent film with enhanced stability in an external environment based on its promising encapsulation properties. These efficient fluorescent films have a bright potential in colour conversion for next-generation display applications.

## 1. Introduction

Over the last few decades, exceptional progress has been attained in formulating phosphors, including rare-earth-ion- or transition-metal-ion-activated phosphors at the micro- or nanoscale, due to their promising wide applications in commercial phosphor-converted white-light-emitting diodes (pc-WLEDs), back-light displays, or high-definition micro-light-emitting diode (micro-LED) displays [1,2,3]. Initially, white LED (WLED) introduced by Nichia used yellow phosphors (YAG: Ce) combined with blue LED, resulting in white light with a low colour rendering index (CRI) (in the range of 60–70) and poor colour gamut. Incorporating red phosphor resulted in a higher CRI (>80) [4]. Some of the common phosphors used for different colour emissions include Ca_1−x_Sr_x_S:Eu^2+^, Sr_2_Si_5_N_8_:Eu^2+^ for red emission, SrGa_2_S_4_:Eu^4+^ for green emission, LiCaPO_4_:Eu^2+^ for blue emission, etc. [5], with rare-earth elements such as cerium and europium and their ions being widely adopted [6]. On the other hand, the report indicates lifetime stability and efficiency issues with phosphor-based materials along with limited access to the raw material and expensive manufacturing costs with a threat to the environment [7,8]. In recent years, to resolve this issue, fluorescent-based organic materials and quantum dots (QDs) have attracted much attention, exhibiting a higher efficiency and a wide colour gamut, along with high CRI [9,10]. QD-based LEDs show benefits such as a narrow emission linewidth, high photoluminescence quantum yield (PLQY > 90%), high photostability, and cost-effectiveness; however, toxic cadmium (Cd)-based QDs demonstrate major disadvantages that can cause harm to humans and the surrounding environment [11,12,13,14]. Another major drawback of QD-based fluorescent materials is their instability and aggregation over time, resulting in a thicker fluorescent film, which might not be a promising material for next-generation display technologies, such as micro-LED. Additionally, for colour conversion applications, the conversion efficiency of the fluorescent film should be high, whereas, in the case of QD-based colour conversion film, it exhibits a lower conversion efficiency. Another drawback of the QD-based emissive film is that it is relatively expensive. For its application as a conversion film, the QD deposition and patterning are very time-consuming and highly rely on the surface modification of the film [15]. On the other hand, conjugated polymer–quantum dots have been used as a conversion film, but the ligands and surfactants related to the QDs generally slow down the energy transfer, resulting in the degradation of the colour conversion properties [16].

For a promising LED technology, a set of red (R), green (G), and blue (B) emitters with enhanced and remarkable high luminous efficiency, stability, and environmental sustainability is a must, as the RGB sub-pixel technology is commonly used in colour reproduction [17,18]. An immense amount of research is being undertaken on the evolution of primary colour emitters composed of red, green, and blue (RGB) [18,19], with red and green fluorescent emitters still requiring enhancement in electroluminescent properties, especially to meet the requirements of next-generation display applications [20,21,22]. In recent years, commercial LEDs based on inorganic phosphors have shown some major drawbacks due to emission scattering from micron-sized phosphor materials and significant reabsorption losses in phosphors, resulting in diminished light output from the overall structure [18,19,23]. In the race to become the ideal precursor, organic dyes have drawn significant attention due to their exciting properties, such as their powerful light-harvesting ability, high extinction coefficient, enhanced photophysical properties, and ease of molecular modification [19]. However, the latest organic light-emitting diode (OLED) technology based on different kinds of organic dyes suffers severe degradation due to various external causes, mainly dust particles, water vapour, and oxygen molecules at room temperature [23]. In addition, LEDs based on inorganic and organic materials suffer internal energy losses [24]. Another promising material, inorganic quantum dots, is highly vulnerable to moisture, UV light, and thermal heating. Primarily, multiple sheets of the barrier are implemented to protect them, making the overall film thick (~300 µm) and expensive [24,25]. In our research, addressing the major device degradation factors such as poor humidity and moisture resistance in the external environment, along with the stability of the quantum yield of the fluorescent material for next-generation display technology, the concept presented here mainly focuses on using an encapsulant polymeric host material, polyvinyl butyral (PVB), to enclose organic dyes in a particular solvent, which results in the fabrication of a fluorescent thin film demonstrating a high and stable quantum yield along with enhanced hydrophobicity, providing its strong degradation resistance to the external environment and room temperature humidity and moisture conditions. Maintaining its high optical performance with time, overall, it presents its candidature of being the primary colour candidate in LEDs. Being an excellent red emitter, we adopted a dicyanomethylene-4H-pyran (DCM) derived 4-(dicyanomethylene)-2-*tert*-butyl-6-(1,1,7,7-tetramethyljulolidyl-9-enyl)-4*H* pyran (DCJTB), developed by Kodiak due to its high photoluminescent quantum yields [20,26], which has been widely used as a red dopant and a host material [27,28], and as a red fluorescent dye [29], and coumarin-6 (3-(2-benzothiazolyl)-7-(diethylamino) coumarin,3-(2-benzothiazolyl)-N,N-diethylumbelliferylamine) was chosen as a green colour precursor. The organic dyes were well mixed with PVB in a dissolving organic solvent tetrahydrofuran (THF) [30] to produce a highly efficient red and green colour. This highly fluorescent solution was further spin-coated on a glass substrate for film formation. The solvent media in which the polymer and dye are embedded plays a vital role in determining the luminescence efficiency.

Being waterproof with high solubility and great adhesive nature, PVB as the host material, along with DCJTB and coumarin-6 in a particular solvent, resulting in the red and green film with uniform thickness, free of heavy metals and rare-earth elements. We measured the quantum yield (QY) of the formed film and achieved a QY (%) as high as 89%, which shows its potential of exhibiting a high QY stability in the film state, whereas, for coumarin-6, the film exhibited a high quantum yield of 80.1%. Both of them displayed a promising quantum yield, which is higher than the Cd-based QDs, with a quantum yield of 52% and 74% for the red and green films [31] and a value of 30% for red emission based on carbon dots used for micro-LED application [32]. Moreover, the refractive index difference between the PVB polymer (1.485) and the THF solvent (1.407) can cause a slight increase in the radiative rate, resulting in an enhanced quantum yield [33]. Additionally, the concentration effect on the quantum efficiency was further investigated, and the impact of PVB on the absorption and quantum yield in relation with film thickness was evaluated. At the molecular level, the bonding mechanism of DCJTB and coumarin-6 with PVB in the solvent was further investigated using Fourier transform infrared spectroscopy (FTIR). A highly efficient and readily stable red and green fluorescent film such as this can be a potential material for colour conversion applications in the mass production of micro-LED displays, overcoming the issue of the mass transfer of millions of RGB micro-LEDs, and can protect from various environmental harm caused by rare-earth metal and heavy metal use. Additionally, by using these thin, uniform fluorescent films and GaN-based LEDs, a better optical performance as a full-colour LED can be achieved.

## 2. Materials and Methods

**Figure 1:**
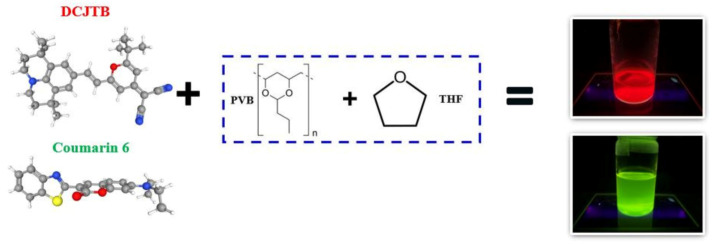
Schematic diagram of the formulation of the hybrid material for the red and green conversion film.

Initially, the glass vial was washed using acetone, isopropanol (IPA), deionised (DI) water, and dried using N_2_ gas. Once cleaned, 10 mL of THF was added as a solvent medium, followed by the addition of a suitable amount (varied between 3 mg to 7 mg) of fluorescent dye DCJTB or coumarin-6 and stirred uniformly for a couple of minutes. Next, 1.5 gm of PVB powder was added as the host material in the prepared solution, along with a magnet stirrer. Once the solution configuration was completed, the solution was stirred at 450 rpm for 24 h to achieve a homogenous solution with no precipitation, as shown in Figure 1. The glass substrates were pre-cleaned for the thin film formation using acetone, IPA, and DI water. After cleaning, the glass substrate was placed on the spin coater machine, and the formed fluorescent solution was deposited across the glass substrate. The spin coating was set at an initial speed of 2500 RPM for 10 s, followed by 6000 RPM for 40 s to form a uniform and homogenous film. Once the film was formed, it was kept on a hot plate at 90 °C for 30 min to evaporate the excess solvent, and the film dried well. Furthermore, the film was subjected to an integrating sphere to calculate its quantum yield. The absorption and excitation spectra were measured using a commercial 460 nm blue LED at current and voltage values of 1 A and 5 V, respectively. The irradiation time of the light source was 75 ms. Furthermore, UV spectroscopy was used to examine the transmittance and reflectance of the fluorescent film in the range of 300 nm–800 nm using a JASCO V770 spectrophotometer (JASCO, Tokyo, Japan). Scanning electron microscopy (SEM) images were taken using 10 kV field emission on a JEOL JSM-7800F microscope (JEOL, Tokyo, Japan) to examine the thickness of the film. Additionally, FTIR of the red and green fluorescent film was performed using Perkin Elmer Spectrum 100 (PerkinElmer Life and Analytical Sciences, Beaconsfield, UK) to analyse the functional groups’ resonance position and the bonding mechanism with the fluorescent dye.

## 3. Results

### 3.1. Fluorescent Film

#### 3.1.1. Quantum Yield (QY) and Molecular Dynamics

**Figure 2:**
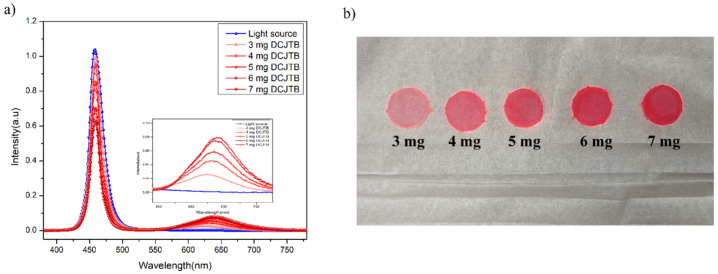
(**a**) Quantum yield profile of DCJTB-based fluorescent film at different concentrations and (**b**) film morphology of DCJTB at different concentrations.

**Figure 3:**
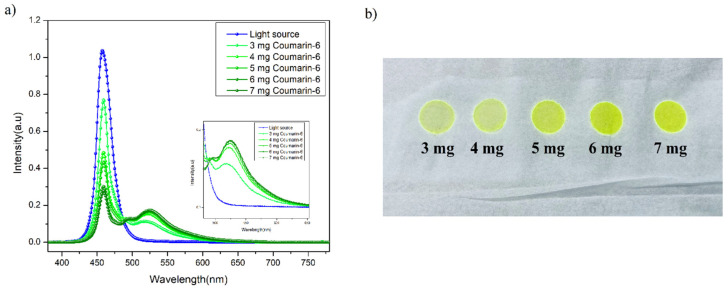
(**a**) Quantum yield profile of coumarin-6-based fluorescent film at different concentrations and (**b**) film morphology of coumarin-6 at different concentrations.

**Figure 4:**
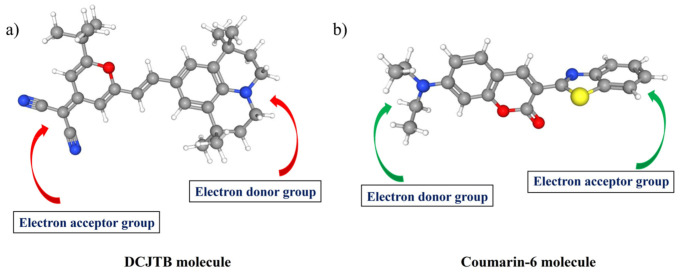
(**a**) The chemical structure of DCJTB and (**b**) the chemical structure of coumarin-6, with blue, red and yellow balls representing N, O and S atoms, respectively.

Fluorescence QY is commonly defined as the ratio of the number of photons emitted to the number of photons absorbed by the sample [34]. In our experiment, QY (%) is generally calculated via the “Direct Excitation method” with the help of an integrating sphere. The QY (%) of the red sample exhibits a maximum quantum yield of 89%. In the other set of experiments, different amounts of DCJTB were taken to study its effect on the quantum yield. It was found that after a certain amount of powder concentration, the quantum yield started to decrease, which can be attributed to concentration quenching phenomena [35,36]. Generally, in an inorganic fluorescent material, if the doping concentration of the activator exceeds a specific value or high doping concentration results in shorting the distance between activators, which is energy transferred between the activators without emitting light, thus leading to reduced luminous efficiency. In addition, the concentration quenching of fluorescent dyes is mainly because excited state fluorescent molecules generate active dimers (excimers) with other ground state fluorescent molecules through the attraction of van der Waals forces or intermolecular forces. The active dimer itself emits light, but it typically has a low quantum efficiency and a relatively small energy gap when the doping concentration of the dye is increased after a specific value (5 mg of DCJTB in our case); the dye molecules are easily stacked to increase the probability of forming active dimers, resulting in a decrease in fluorescence efficiency [36]. Another aspect of concentration quenching is the decrease in the separation of the chromophores distance with an increase in the concentration, which enhances the non-radiative de-excitation routes and results in diminished quantum yield emission [37], which can be seen in coumarin-6 in our case. The concentration mechanism can have various interaction pathways depending on the host material and the environment. The quantum yield plot and film morphology for different concentrations of DCJTB and coumarin-6 are shown in Figure 2a,b and Figure 3a,b, respectively. Interestingly, with an increase in the concentration of the organic precursor, a red shift in the emission wavelength was observed, as seen in Table 1 and Table 2. This red shift can be attributed to the increase in the absorption of the fluorescent radiation of a shorter wavelength within the film, with an increase in the dye concentration. Consequently, the fluorescence emission in this range declines and becomes richer in the red, resulting in the shift of the fluorescence peak towards a longer wavelength [38]. Next, looking from the molecular level, in principle, these organic molecules (DCJTB and coumarin-6) contain an electron-donating group and an electron-withdrawing group as donor and acceptor moieties, commonly called push–pull dyes. In the case of coumarin-6, the electron-donating groups, i.e., diethylamino group, is at the (C7) position, whereas the electron-withdrawing group, i.e., 1,3-benzothiazol-2-yl, is at the (C3) position via resonance and inductive effects [39], as shown in Figure 4b. On the other hand, in the DCJTB molecule, the highest occupied molecular orbit electron-withdrawing localized on the julolidine moieties acts as an electron donor group, whereas the lowest unoccupied molecular orbital (LUMO) levels are on the dicynovinyl moieties, acting as an electron acceptor groups [40], as shown in Figure 4a. This unique configuration makes these molecules have a small ΔE_st_ between their singlet (S_1_) and triplet (T_1_) excited state, which increases the T_1_ → S_1_ reverse intersystem crossing, making them exhibit thermally activated delayed fluorescence (TADF), resulting in a higher quantum yield [41]. The obtained quantum efficiency and enhanced stability in the external environment ensure its potential as a colour conversion layer for its practical application in full-colour displays.

#### 3.1.2. Optical and Morphological Characteristics

**Figure 5:**
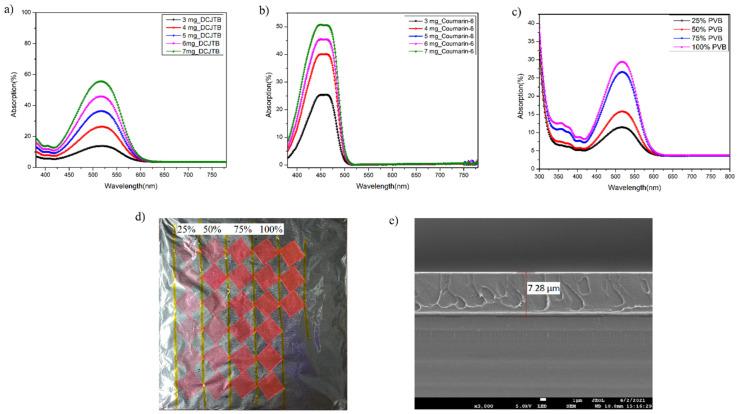
(**a**) Absorption profile at varied concentrations of (**a**) DCJTB, (**b**) coumarin-6, (**c**) absorption profile of red fluorescent film at different PVB concentrations, (**d**) optical appearance of the fluorescent film with variation in PVB concentration, and (**e**) cross-sectional image of the fluorescent film taken under SEM.

UV spectroscopy was performed on the formed fluorescent film. Figure 5a shows the absorption spectra of the red fluorescent film. The maximum absorption peak was found to be at 518 nm for the film [42,43,44]. On the other hand, the absorption spectra of the green fluorescent films are shown in Figure 5b, which exhibited a maximum absorption at 456 nm. Additionally, the film thickness was investigated using a scanning electron microscope, and the cross-sectional view of the film shows a thickness of 7.28 µm, as seen in Figure 5e.

**Figure 6:**
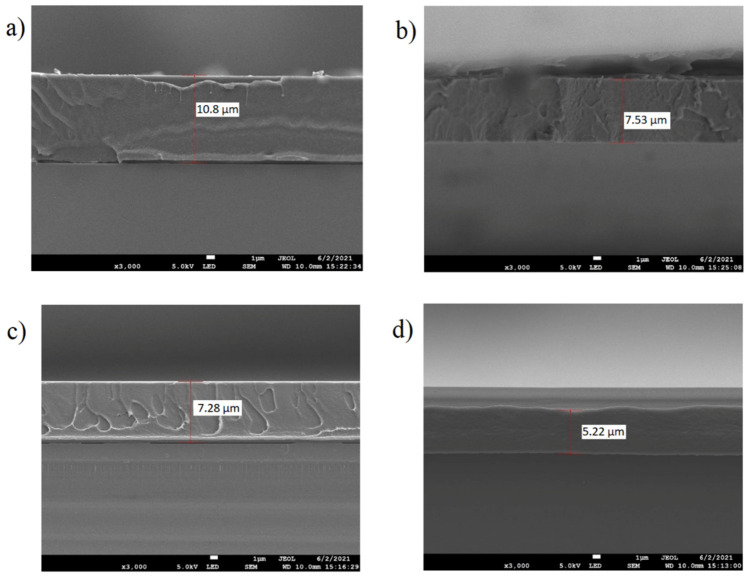
Spin speed-dependent thickness and quantum yield (**a**) 2500–4000 RPM, (**b**) 2500–5000, (**c**) 2500–6000, and (**d**) 2500–7000.

Next, to check the optimized concentration of PVB and its effect on the thin film optical properties and thickness, the PVB concentration was varied, being 25% (0.375 gm), 50% (0.750 gm), 75% (1.125 gm) and 100% (1.5 gm), with the dye and organic solvent concentration fixed. In principle, the thickness of the fluorescent film highly depends on the viscosity of the fluorescent solution. In our case, the viscosity was altered by adding PVB polymer and changing its concentration, and it was found to be an effective way to tune the thickness of the film. Figure 5c shows the effect of PVB concentration on the thin film’s absorption (%) capability. It was found that with an increase in the concentration of PVB, the absorption (%) of the fluorescent film increased from 11.4% for 0.375 gm of PVB to 29.4% for 1.5 gm of PVB, which can be further observed by the optical appearance of the red fluorescent film, as shown in Figure 5d. It can be seen that with an increase in the concentration of PVB, the film exhibited a pure red colour, demonstrating better binding and enhancing the optical performance characteristics of PVB with the dye. Furthermore, the effect of spin speed on the quantum yield and film thickness was evaluated, as these parameters are crucial for a colour conversion film when amalgamating with blue LED for full-colour display applications. It was found that with an increase in the RPM speed, there was a significant decrease in the thickness of the film and an increment in the quantum yield due to the reduction of the internal absorption with a decrement in the thickness, as presented in Figure 6 and Table 3. Moreover, the photostability of the fluorescent film was investigated in an inert system under UV exposure of intensity 1.3 W/cm^2^, which is over 200 times larger than the UV intensity of sunlight (energy in the UV range, generally <400 nm, times the sunlight intensity of 100 mW/cm^2^). For coumarin-6, the sample’s QY (%) was calculated for different hours of UV exposure (0 h, 2 h and 4 h). The 6 mg coumarin-6-based film was adopted to check the photostability, whose QY (%) calculated on day 1 of the formation is mentioned in the above section. Keeping the sample at normal room temperature and humid conditions for 30 days, the photostability experiment was conducted, where the sample was exposed under UV light for different time periods. At the beginning of the experiment, the QY (%) was measured to be 65.04%, and after 2 h of continuous exposure, the QY (%) was again measured, and it was found to be 64.32%. After this, the sample was exposed for a further 4 h, and the QY (%) was measured to be 62.80%, as mentioned in Table 4. Apart from the photostability of the film, this table also shows the quantum yield stability of the fluorescent film over a period of 30 days, indicating its robustness in external environmental conditions.

#### 3.1.3. PVB- and PVP-Based Hydrophilicity

**Figure 7:**
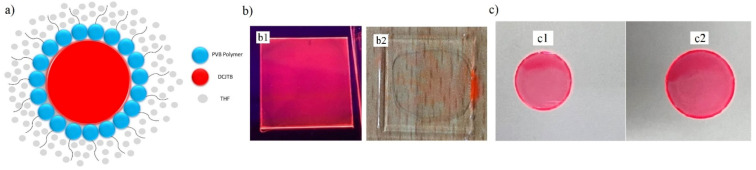
(**a**) PVB binding mechanism with the organic dye in the hybrid material system, (**b**) PVP-based red fluorescent film’s hydrophilicity: b1 and b2 represent the film before and after dipping into the water, respectively, and (**c**) PVB-based red fluorescent film’s hydrophobicity: c1 and c2 represent the film before and after dipping into the water, respectively.

**Figure 8:**
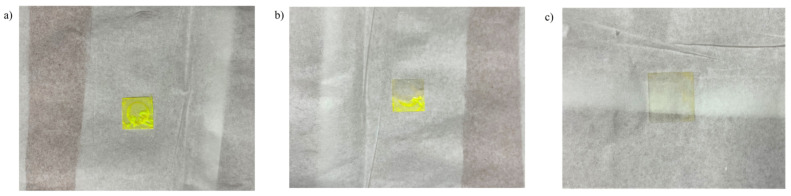
PVP-based green fluorescent film: (**a**) morphology after film formation, (**b**) partial immersion in water, and (**c**) complete immersion of the fluorescent film in water.

PVB polymer is widely adopted for composite formation [45] and various polymer films [46] due to its attractive properties, such as more vital binding ability, sharper optical clarity, and flexibility provided to its end product. Generally, the PVB structure contains hydrophilic vinyl alcohol and hydrophobic vinyl butyral groups, which act as promoters of polymer adhesive and as a binder for organic moieties [45]. Furthermore, PVB is mostly polyhydroxy and polyacetal, due to the presence of butyral and hydroxyl in major proportions with a lesser amount of acetyl groups, and shows a higher dispersing ability to dyes. Due to the presence of an anchoring group and long carbon chain which can stretch in the solvent, a hindrance layer for the colloidal solution is formed, as presented in Figure 7a, resulting in stable dispersion in the solvent. In our experiment, we preferred a PVB-based spin-coated polymer film in THF solvent. PVB-containing films prepared in THF are continuous and homogenous, depending on the solvent–polymer and solvent–substrate interaction energy [47]. On the other hand, polyvinyl pyrrolidone (PVP) is a polymer with unusual binding properties [48] and greatly shows hydrophilicity. When interacting with water, PVP binds with a water molecule and is held with H_2_O via hydrogen bonding to the C=O group on the pyrrolidone ring [49], while PVB is bound with water near the carbonyl group and the C–N bonds [50]. In our experiment, we found that the PVP-based red fluorescent film [51], once exposed to water, dissolves easily within a couple of minutes, showing its hydrophilicity ability and hindering its use in many practical applications. In contrast, the hydrophobic PVB-based film sustained its original homogenous form even after being exposed to water Figure 7b,c. After DCJTB, coumarin-6 was tested for PVP polymeric host and its water resistance and film morphology, as shown in Figure 8. Figure 8a shows a non-uniform green emissive film based on PVP polymer, which was further subjected to water, and after a couple of minutes, the film was thoroughly washed away, as seen in Figure 8c. This result clearly shows that PVP is not suitable as a host material for fluorescent film formation, as it is highly hydrophilic in nature, making it unsuitable for the external environment.

#### 3.1.4. FTIR Spectroscopy

**Figure 9:**
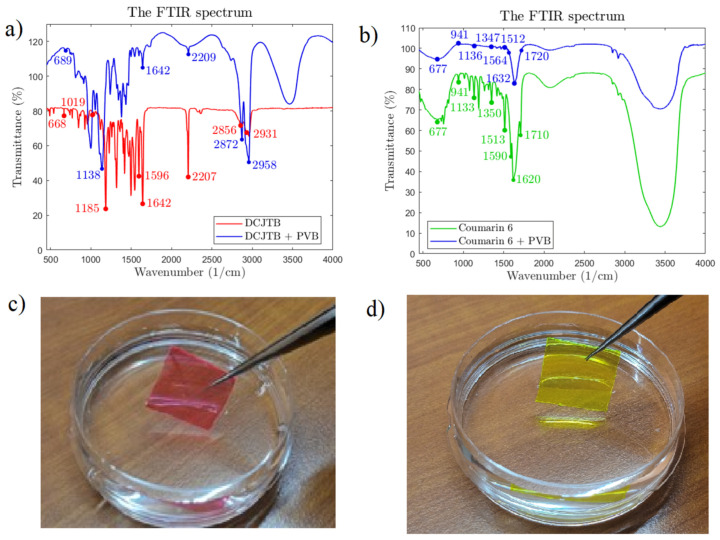
FTIR spectroscopy: (**a**) pure DCJTB and hybrid material, (**b**) pure coumarin-6 and hybrid material, (**c**) red hybrid material in water, and (**d**) green hybrid material in water.

In principle, once the molecule absorbs light energy, the frequency of the molecular vibration increases and transits from the ground state to the excited state. The absorption of the infrared light results in the vibration of the molecules’ polar part, which creates the base to examine the molecular structure, different functional groups, and chemical bonding of the organic fluorescent dye with the host in the solvent in our experiment. FTIR was conducted to evaluate the absorption position of the various characteristic functional groups in the organic fluorescent material DCJTB and coumarin-6 in specific solvent dichloromethane (DCM) in the range 450–4000 cm^−1^. DCJTB is an organic fluorescent dye-containing pyran and julolidine moieties with a nitrile group (C≡N) as a chromophore responsible for the colour of the dye [52]. As seen in Figure 9a for DCJTB, there is a strong and sharp peak at 2207 cm^−1^ due to −C≡N and the stretching vibration of the cyano group, the chromophore in the DCJTB molecule. Another peak at 668 cm^−1^ corresponds to C–H out-of-plane bending. A sharp and strong peak at 1185 cm^−1^ can be assigned to the C–C band and C–H stretching modes of the CH_3_ and CH_2_ groups. In contrast, a major peak at 1642 cm^−1^ can be attributed to the C=C stretching band and antisymmetric stretching band, the H–O–H bending of water. The red dye also shows a couple of weak peaks at 2856 cm^−1^ and 2930 cm^−1^, which can be assigned to the symmetrical and asymmetrical stretching vibration of the methylene group (CH_2_), respectively [44]. On the other hand, Figure 9b shows that the FTIR for coumarin-6 dissolved in DCM exhibits a significant peak at 1615 cm^−1^, with two shoulder peaks at 1589 cm^−1^ and 1711 cm^−1^, corresponding to the C=C stretching, C=N stretching, and C=O stretching, respectively. Another peak at 1512 cm^−1^ can be assigned to C–C stretching, a prominent peak at 1349 cm^−1^ corresponds to C–N stretching, whereas significant peaks at 1192 cm^−1^, 1133 cm^−1^, and 1077 cm^−1^ are attributed to C–H in-plane bending. Furthermore, C–H out-of-plane bending is found at 941 cm^−1^, with smaller peaks at 818 cm^−1^, 755 cm^−1^, and 728 cm^−1^. Another smaller peak at 677 cm^−1^ is attributed to C–S stretching [39]. In coumarin-6, the thiazole group is the chromophore with an original peak of C–S at 677 cm^−1^, C–N at 1350 cm^−1^, and C=N at 1590 cm^−1^, which after mixing with PVB, resulted in a peak at 677 cm^−1^, 1347 cm^−1^, and 1564 cm^−1^, corresponding to C–S, C–N, and C=N bonds, respectively. Notably, the prominent peaks of the organic fluorescent dyes were essentially unchanged, with a slight peak shift after adding inorganic polymer for the hybrid material formation, as seen in Figure 9a,b. These results reveal that the polymeric host material PVB merely encloses the fluorescent dye and acts as an encapsulant without forming a new chemical bond and changing the initial properties of the fluorescent precursor. Moreover, comparing the spectrum of hybrid material with the pure PVB spectrum, we can see some significant peaks around 3500 cm^−1^, 2872 cm^−1^ and 2958 cm^−1^ corresponding to the phenolic −OH stretching vibration, asymmetric and symmetric −CH_2_ stretching, respectively, which are generally found in PVB [53]. The encapsulation using PVB is excellent with the hydrophobicity property of the polymeric hybrid material, depicting its promising application under the external environment. The films are not influenced even when being dipped in water, as shown in Figure 9c,d.

## 4. Conclusions

In this article, we presented rare-earth free elements as the primary source to make a red fluorescent film which can be applied as a red colour conversion layer and LED application in the near future. The prepared red and green film displayed high quantum efficiency of up to 89% and 80%, respectively. There was a variation in the quantum yield of the fluorescent film on varying the concentration of the precursor, and it was attributed to the concentration quenching mechanism. PVB, the polymeric host material, further resulted in an enhanced quantum yield, optical clarity and uniformity, along with strong hydrophobicity to the fluorescent films, making them robust to the external environment. Furthermore, the effect of PVB concentration on the absorption and film morphology was studied, and it was found that with an increase in the PVB concentration, the absorption of the film increases significantly, displaying its strong effect on the optical performance of the fluorescent film. Next, the FTIR spectroscopy reveals that the red fluorescent precursor has a nitrile (C≡N) group as a chromophore at a wavenumber of 2207 cm^−1^. After formulating the hybrid material, there was a slight shift in the peak to 2209 cm^−1^, whereas, for the green fluorescent film, the thiazole group being the chromophore group, showed very similar results after mixing with PVB. It was found that with the addition of the host (PVB) or the solvent (THF), there is no new bond formation, and only intermolecular forces are present between them. Finally, this experiment opens a door for formulating eco-friendly, highly efficient, thinner and stable colour conversion film, fulfilling the requirements for advancing next-generation display applications like micro-LEDs.

## Figures and Tables

**Table 1:** Quantum yield and absorption of the different concentrations of DCJTB.

DCJTB Concentration (mg)	Quantum Yield (%)	Absorption (%)	Emission Wavelength (nm)
3	80.1	13.83	629 nm
4	86.5	26.23	632 nm
5	89.0	36.35	634 nm
6	80.9	45.83	639 nm
7	79.4	55.54	641 nm

**Table 2:** Quantum yield and absorption of the different concentrations of coumarin-6.

Coumarin-6 Concentration (mg)	Quantum Yield (%)	Absorption (%)	Emission Wavelength (nm)
3	80.4	25.39	519 nm
4	73.1	40.08	521 nm
5	72.6	45.38	523 nm
6	66.9	45.46	525 nm
7	67.7	50.65	528 nm

**Table 3:** Variation in quantum yield and thickness with the spin speed of the thin film formation.

RPM Speed	Quantum Yield (%)	Thickness (µm)
2500–4000	76.6	10.3
2500–5000	78.7	7.5
2500–6000	79.4	7.2
2500–7000	80.3	5.2

**Table 4:** Photostability of the fluorescent film under UV exposure.

Exposure Time	Quantum Yield (%)
Day 1	66.90
Day 30 (0 h)	65.04
2 h	64.32
4 h	62.80

## Data Availability

Data contained within the article.
